# Survival from testicular cancer in England and Wales up to 2001

**DOI:** 10.1038/sj.bjc.6604597

**Published:** 2008-09-23

**Authors:** U Nur, B Rachet, E Mitry, N Cooper, M P Coleman

**Affiliations:** 1 Cancer Research UK Cancer Survival Group, Non-Communicable Disease Epidemiology Unit, Department of Epidemiology and Population Health, London School of Hygiene and Tropical Medicine, Keppel Street, London WC1E 7HT, UK; 2 Departement of Hépatogastroentérologie et Oncologie Digestive, Centre Hospitalo-Universitaire Ambroise-Paré, 9 avenue Charles de Gaulle, F-92100 Boulogne, France; 3 Social and Health Analysis and Reporting Division, Office for National Statistics (Room FG/114), 1 Myddelton Street, London EC1R 1UW, UK

Testicular cancer has a peak incidence at around 30 years of age, and it is the most common cancer in men under 35 years of age, accounting for a third of all cancers in that age range (Adami *et al*, 1994). The age distribution is unusual among adult malignancies, with almost 70% of cases arising in men under 40 years of age, and only about 2% in men over 70 years of age. Incidence has increased steadily in many developed countries over the last 50 years, including the United Kingdom (Coleman *et al*, 1993; Richiardi *et al*, 2004). In men under 50 years of age, incidence has risen almost two-fold in England and Wales in the last 30 years, but the increase has been much smaller among older men (Quinn *et al*, 2001).

Age-standardised mortality was 1 per 100 000 per year during the 1950s, and it rose slightly during the 1960s and 1970s. Advances in the treatment of testicular cancer since the 1960s have had a dramatic effect on survival (Coleman *et al*, 1999), and men born during the 1950s and 1960s had substantially lower testicular cancer mortality in the late 1970s and early 1980s than earlier birth cohorts, despite the substantial rise in incidence (Quinn *et al*, 2001).

Cryptorchidism is one of the few known risk factors (Richiardi *et al*, 2002). A positive family history also carries a higher risk, but vasectomy has not been shown to increase risk (Møller *et al*, 1994).

Most testicular cancers are germ cell tumours, either seminoma or non-seminoma. Seminomas arise from the sperm-producing testicular germ cells, whereas non-seminoma germ cell tumours include teratoma, choriocarcinoma and yolk sac tumours. Seminomas are treated by surgical removal of the affected testicle (orchiectomy) and adjuvant radiotherapy. Surgical treatment is also effective with early stage teratomas. Orchiectomy alone cures around 80% of testicular cancer patients (Dearnaley *et al*, 2006).

We analysed survival patterns for 18 605 men diagnosed with testicular cancer aged 15–99 years in England and Wales during 1986–1999 and followed up to 31 December 2001. Less than 5% of tumour records had to be excluded from analysis: 2% because the vital status was not known when data were extracted for analysis, less than 2% because the testicular cancer either followed or was synchronous with another primary malignancy, and about 1% because the date of diagnosis was the same as the date of death (‘zero survival’). Virtually all the tumours in the last category will have been registered solely from a death certificate, from which the date of diagnosis is unavailable, as very few men would be expected to have died on the day of diagnosis, but the two categories could not be reliably distinguished in the national data.

In total, 54% of tumours were seminomas, and 29% teratomas, similar to the proportions seen in the late 1980s (50 and 30%). Rare morphologic types included choriocarcinoma, trophoblastic teratoma (1%), Leydig cell tumour (0.3%) and a few Sertoli cell tumours. Although cryptorchidism is a known risk factor, less than 1% of tumours were recorded as having arisen in an undescended testis.

## Survival trends

**Figure 1:**
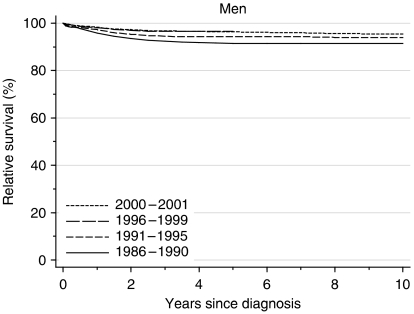
Relative survival (%) up to 10 years after diagnosis by calendar period of diagnosis: England and Wales, adults (15–99 years) diagnosed during 1986–1999 and followed up to 2001. Survival estimated with cohort or complete approach (1986–1990, 1991–1995, 1996–1999) or hybrid approach (2000–2001) (see Rachet *et al*, 2008).

For men diagnosed during 1986–1990, relative survival was 96% at 1 year after diagnosis and 91% at both 5 and 10 years. Survival has risen further with time, so that for men diagnosed during 1996–1999, relative survival had reached 98% at 1 year after diagnosis and 97% at 5 years (Table 1, Figure 1). Ten-year survival was effectively the same as 5-year survival.

After adjustment for deprivation, the average increase every 5 years was 0.5% for 1-year survival and 1% for 5-year survival. These trends are not statistically significant, but the small increases in 1-, 5- and 10-year survival were consistent across three time periods.

Short-term prediction of survival up to 10 years, derived from hybrid analysis (Brenner and Rachet, 2004) of the conditional survival probabilities actually observed among men alive at some point during 2000–2001, suggests that survival will not increase much further in the near future (Table 1). If the most recently observed survival patterns were to persist unchanged for the rest of the decade, men diagnosed in the first few years of the 21st century may expect 10-year relative survival rates that approach 96%.

Survival rose substantially for older men during the 1990s, and the huge decline in survival with age became less marked. Thus, 5-year relative survival was over 90% for men diagnosed under the age of 50 during the decade 1986–1995, but less than 70% for men aged 70–79 years, and less than 40% for elderly men (80–99 years). For men aged 80–99 years diagnosed during 1996–1999, however, 5-year survival had risen to 55% (data not tabulated).

## Deprivation

**Figure 2:**
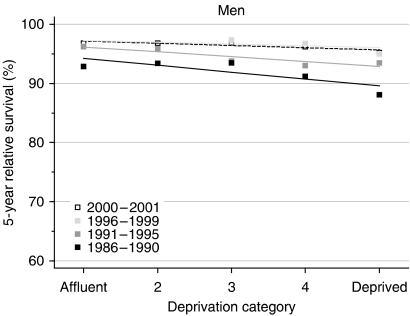
Trends in the deprivation gap in 5-year relative survival (%) by calendar period of diagnosis: England and Wales, adults (15–99 years) diagnosed during 1986–1999 and followed up to 2001.

For men diagnosed during 1986–1990, relative survival up to 10 years after diagnosis was significantly lower, by 3–5%, among those in the most deprived group than those in the most affluent group (Table 2, Figure 2). By the late 1990s, this deprivation gap in survival at 1, 5 and 10 years had fallen to as little as 1%, and it was only of borderline significance for 1-year survival.

Short-term predictions, based on hybrid analysis of the probabilities observed during 2000–2001, suggest that the deprivation gradient will remain small, of the order of −1% (Table 2).

## Comment

Survival from testicular cancer is the highest for any malignancy among adult males in England and Wales.

The gain in survival over the last 20 years has been little short of remarkable. One-year and five-year survival for men diagnosed in England and Wales during the early 1970s was already 82 and 69%, respectively, but it rose to 95 and 89%, respectively, for men diagnosed in the early 1980s, just a decade later (Coleman *et al*, 1999). The data reported here show that for men diagnosed in the late 1990s, short-term survival was already approaching the theoretical maximum of 100%, and even longer-term survival exceeded 95%, whereas period analysis suggests that the increase is likely to continue, albeit more slowly.

The increase in survival among the oldest men is not statistically significant, and it concerns only a small minority of cases, but it may be that the very high survival attained in younger men has led to treatment of curative intent being offered to even the oldest men with testicular cancer.

Relative survival at 10 years is now virtually identical to that at 5 years, which itself is already more than 95%. This indicates that, among men who survive 5 years, there is virtually no further excess mortality over that of men in the general population, after adjustment for age and deprivation. In other words, the data provide strong public health evidence that 5-year survivors of testicular cancer are now virtually all ‘cured’, because, taken as a group, their mortality is no different from that of their peers in the general population.

Coherence of national trends in incidence, survival and mortality since the 1970s is striking. Despite rapidly rising incidence, survival has risen and mortality has continued to fall. This provides convincing evidence of the nation-wide impact of the addition of platinum-based chemotherapy in the 1970s to early diagnosis, surgery and radiotherapy.

Despite the fact that relative survival was very high for men in all socioeconomic groups combined, there was still a significant socioeconomic gradient in survival. This is so even after adjustment for socioeconomic differences in the background risk of death by the use of life tables that are specific for each socioeconomic group.

The trend in overall survival now approaches 100%, and the deprivation gap in survival has fallen commensurately, from 13% or more for men diagnosed during the early 1970s to less than 5% for men diagnosed during the late 1990s. This narrowing of the deprivation gap appears to reflect a ‘ceiling’ effect, as there is little room for any further increase in relative survival among the most affluent group. Survival for the most affluent men diagnosed most recently approaches 100%.

Despite the unquestionably dramatic improvement in survival, men in the most deprived categories still only attain the relative survival of the most affluent men with a time lag of at least 5 calendar years.

## Figures and Tables

**Table 1:** Trends in relative survival (%) by time since diagnosis and calendar period of diagnosis: England and Wales, adults (15–99 years) diagnosed during 1986–1999 and followed up to 2001

		**Calendar period of diagnosis^a^**						**Average change (%)**		**Prediction^c^ for patients**	
		**1986–1990**		**1991–1995**		**1996–1999**		**every 5 years^b^**		**diagnosed during 2000–2001**	
**Time since diagnosis**		**Survival (%)**	**95% CI**	**Survival (%)**	**95% CI**	**Survival (%)**	**95% CI**	**Survival (%)**	**95% CI**	**Survival (%)**	**95% CI**
1 year	Men	**95.7**	(95.1, 96.3)	**97.2**	(96.8, 97.6)	**98.1**	(97.7, 98.4)	**0.5**	(−0.3, 1.3)	**98.3**	(97.7, 98.7)
5 years	Men	**91.4**	(90.6, 92.2)	**94.3**	(93.6, 94.8)	**96.5**	(95.9, 97.0)	**1.2**	(−0.2, 2.5)	**96.3**	(95.5, 97.0)
10 years	Men	**91.4**	(90.6, 92.2)	**94.0**	(93.3, 94.6)			**2.0**	(−0.5, 4.4)	**95.5**	(94.5, 96.3)

CI=confidence interval.

a Survival estimated with cohort or complete approach (see Rachet *et al*, 2008).

b Mean absolute change (%) in survival every 5 years, adjusted for deprivation (see Rachet *et al*, 2008).

c Survival estimated with hybrid approach (see Rachet *et al*, 2008).

**Table 2:** Trends in the deprivation gap in relative survival (%) by time since diagnosis and calendar period of diagnosis: England and Wales, adults (15–99 years) diagnosed during 1986–1999 and followed up to 2001

		**Calendar period of diagnosis^a^**						**Average change** (%)		**Prediction^c^ for patients**	
		**1986–1990**		**1991–1995**		**1996–1999**		**every 5 years^b^**		**diagnosed during 2000–2001**	
**Time since diagnosis**		**Deprivation gap (%)**	**95% CI**	**Deprivation gap (%)**	**95% CI**	**Deprivation gap (%)**	**95% CI**	**Deprivation gap (%)**	**95% CI**	**Deprivation gap (%)**	**95% CI**
1 year	Men	−2.7**	(−4.3, −1.0)	−1.5**	(−2.6, −0.5)	−1.1*	(−2.1, −0.1)	**0.8**	(−0.2, 1.8)	**−0.9**	(−2.1, 0.3)
5 years	Men	−4.7**	(−7.0, −2.4)	−3.3**	(−5.0, −1.7)	**−1.3**	(−3.3, 0.8)	1.8*	(0.2, 3.4)	**−1.5**	(−3.7, 0.6)
10 years	Men	−4.3**	(−6.6, −1.9)	−3.6**	(−5.3, −1.8)			**0.7**	(−2.2, 3.6)	**−1.2**	(−3.8, 1.4)

CI=confidence interval.

a Survival estimated with cohort or complete approach (see Rachet *et al*, 2008).

b Mean absolute change (%) in the deprivation gap in survival every 5 years, adjusted for the underlying trend in survival (see Rachet *et al*, 2008).

c Survival estimated with hybrid approach (see Rachet *et al*, 2008).

^*^P<0.05; ^**^P< 0.01.
